# Feasibility of the Omaha system for the care of children with dilated cardiomyopathy

**DOI:** 10.3389/fped.2023.1136663

**Published:** 2023-05-30

**Authors:** Qin Zhang, Ai Zhang, Yanqin Wang, Tiewei Lv, Ping Sun, Xiaoxia Zhao, Rui Li, Xianlan Zheng

**Affiliations:** ^1^ Department of Cardiology, Chongqing Key Laboratory of Pediatric, Ministry of Education Key Laboratory of Child Development and Disorders, National Clinical Research Center for Child Health and Disorders, Children’s Hospital of Chongqing Medical University, Chongqing, China; ^2^ Department of Nursing, Chongqing Key Laboratory of Pediatric, Ministry of Education Key Laboratory of Child Development and Disorders, National Clinical Research Center for Child Health and Disorders, Children’s Hospital of Chongqing Medical University, Chongqing, China

**Keywords:** Omaha system, dilated cardiomyopathy, children, nursing language, continuous nursing care

## Abstract

**Aim:**

To explore the feasibility of Omaha system theory in the care of children with dilated cardiomyopathy (DCM), which may provide a practical basis for the continuous nursing of DCM children.

**Methods:**

A total of 1,392 records describing symptoms, signs, and nursing interventions were extracted from the medical records of 76 children suffered from DCM. Content analysis method was used to find out existent nursing problems, make precise nursing plans, and take corresponding nursing measurements according to the medical records of DCM children. Cross-mapping method was utilized to compare the conceptual consistency of the medical records and Omaha system (problem classification and intervention subsystems).

**Results:**

Of the total 1,392 records, 1,094 (78.59%) were complete consistency, while 245 (17.60%) were partial consistency, and 53 (3.81%) were inconsistency with the Omaha system concepts. The concept matching degree of medical records and Omaha system was approximately 96.19%.

**Conclusions:**

The Omaha system may be an effective nursing language for Chinese DCM children, which may be useful to guide nurses in the care of DCM. Further well-design studies need to fully evaluate the feasibility and effectiveness of the Omaha system in nursing children with DCM.

## Introduction

Childhood cardiomyopathy, an important cause of death in children, with an incidence of 1:100,000 person-years (1). Dilated cardiomyopathy (DCM) is a myocardial disease characterized by left ventricular dilatation and systolic dysfunction, commonly resulting in congestive heart failure, and is the most common form of cardiomyopathy and the cause of heart transplantation in children (2, 3). Approximately 50% of childhood cardiomyopathies are reported to have DCM, which a 5-year rate of death or transplantation is up to 30%–50% (4). DCM seriously affects the physical and mental health of children and also imposes a heavy mental and economic burden on families and society.

At present, clinical medication and symptomatic treatments are main protocols for DCM children (1). Evidences have showed that suitable care modes can benefit heart-related disease children in terms of treatment and prognosis (5, 6). The Omaha system is a standardized nursing terminology for nursing practice recognized by the American Nurses Association, which consists of the problem classification scheme, intervention scheme, and problem rating scale for outcomes (7). It has been widely used in the field of nursing research and education (8, 9). Fang et al. reported the community rehabilitation nursing of Omaha system in stroke patients with previous falls (10). Yin et al. explored the effects of Omaha system-based continuing care on the medication compliance, quality of life, and prognosis of coronary heart disease patients after percutaneous transluminal coronary intervention (11). However, the application of Omaha system is primarily in adult care (10–12). Research by Zhuang et al. assessed roles of Omaha System on the psychological status, self-esteem, and quality of life in epileptic children (13). To the best of our knowledge, the impact of Omaha system on continuous nursing care of DCM children remains unclear.

Herein, we compared the conceptual consistency of the medical records and Omaha system to explore the feasibility of Omaha system for continuous nursing care in DCM children, which may provide a reference for continuous, dynamic, standardized nursing care in Chinese children with DCM.

## Methods

### Study design and participants

The medical records of children with DCM were collected from the Cardiovascular Department of Children's Hospital of Chongqing Medical University between January 2017 and December 2020 in this retrospective study. The diagnostic criteria of DCM were the 2019 American Heart Association (AHA) (1). Children who had incomplete, or missing nursing records were excluded. The common nursing problems and nursing measures of DCM children were shown in Supplementary Tables S1, S2. This study was approved by the Ethics Committee of Children's Hospital of Chongqing Medical University [(2020) No.57]. The informed consents were provided by their guardians.

### Omaha system measurement

The Omaha system includes the problem classification scheme, intervention scheme, and problem rating scale for outcomes. The problem classification scheme consists of 42 health problems in four fields: environment, psychology, physiology, and health-related behavior. The intervention scheme includes health education, guidance and counseling, treatment and procedure, and case management and monitoring. The problem rating scale for outcomes has three areas of cognition, behavior, and condition, and is evaluated by Likert 5 hierarchical digital measurement. From the perspective of holistic nursing, the Omaha system comprehensively evaluates patients’ existing or potential nursing problems and adopts targeted nursing intervention and effect evaluation, which can provide practitioners with a patient-centered and clear holistic nursing thinking mode. The Chinese version of the Omaha system proposed by Professor Huang et al. was used in this study (14). Its reliability coefficient, Cronbach's *α* coefficient, was 0.729 and Content validity (CVI) was 0.85, indicating its good reliability and validity.

The Omaha problem classification and intervention systems were used in this study. The forms were finalized after review by one deputy chief nurse and one chief physician. Two members of the research group searched the electronic disease record system of the hospital, and extracted the description of nursing evaluation, nursing diagnosis, nursing plan, and intervention measures for DCM children as analysis items. Content analysis method, cross-mapping method (15) were used to assess Omaha problem classification scheme and intervention scheme. Repeated care descriptions for the same child were recorded only once. The conceptual consistency degree of the medical records and Omaha system is divided into three levels: sufficient consistency = 3, indicating the textual description or semantic consistency; partial consistency = 2, indicating partial consistency of the concepts, such as the detailed description, while the Omaha system concept is relatively broad or narrow; inconsistency = 1, indicating that the analysis item is different or there is no relevant item, and the researchers need to supplement it in the “Other” column.

### Quality control

Before starting the study, 20% of the medical records were selected by the convenience sampling method for the evaluation of the internal consistency of the study results (16). According to the research methods, the consistency analysis of relevant content was carried out independently by 2 uniformly trained nursing postgraduates. For differences, researchers will consult nurses and doctors with senior professional titles in pediatric cardiovascular specialty, and then decide on the anastomosis degree after discussion with the members of the research group. The reliability of the results was analyzed to calculate the Kappa coefficient. The analysis showed that the Kappa coefficient was 0.786, 0.815, and 0.75, indicating its good stability (17).

### Statistical analysis

SPSS software (version 17.0, IBM, USA) was used for statistical analyses. Normal distribution data were expressed as the mean ± standard deviation (SD) and analyzed using the independent sample *t*-test. Categorical data were described as number and proportion [*n* (%)], and performed by the Mann-Whitney *U*-test.

## Results

### Characteristics of DCM children

A total of 76 eligible DCM children were included in this study, including 30 males and 46 females. Patients’ age ranged 58 days–16 years, with an average age of (8.05 ± 5.03) years. All children suffered from various degrees of HF, of which 17 (22.37%) were in grade I–Ⅱ, and 59 (77.63%) were in grade Ⅲ–Ⅳ. Fifty-six (73.68%) patients were combined with arrhythmia, and 34 (44.74%) with respiratory infection.

### The conceptual consistency between the medical records and Omaha system in DCM children

Totally 800 items describing the caring methods, symptoms and signs of children with DCM, and 592 items of nursing interventions were extracted from 76 medical records. Table 1 shows the consistency between the medical records and Omaha system in DCM children. There were 644 (80.05%) and 450 (76.01%) sufficient consistency in the caring methods, symptoms and signs of DCM children, respectively.

**Table 1:** Consistency between nursing records and Omaha system in DCM children.

Items	Total	Complete consistency, *n* (%)	Partial consistency, *n* (%)	Inconsistency, *n* (%)
Signs and symptoms	800	644 (80.05)	110 (13.75)	46 (5.75)
Caring methods	592	450 (76.01)	135 (22.80)	7 (1.18)
Total	1,392	1,094 (78.59)	245 (17.60)	53 (3.81)

DCM, dilated cardiomyopathy.

### The conceptual inconsistency between the medical records and Omaha system in DCM children

Of the total 1,392 items, 53 were inconsistent with the Omaha system, where 46 described the caring methods, symptoms and signs of children with DCM, and 7 described nursing interventions, as detailed in Tables 2, 3, respectively.

**Table 2:** Nursing records inconsistent with the Omaha problem classification subsystem (*n* = 46).

Omaha problem classification	Inconsistent with concept description in nursing records	*n*
Environment	Falling, falling from bed	1
Psychology	Energielos, sedation	2
Biology	Water and sodium retention, cardiac insufficiency, enlarged cardiac boundary, precardiac area uplift, high sweating, cold limbs, disruption of milk intaking, groan, feeding interruption, aspiration, bulging anterior fontanelle, etc.	38
Health-related behavior	Excessive salt restriction, feeding difficulties, schooling suspension, repeated hospitalization, dorsal foot artery pulsatile asymmetry	5

**Table 3:** Nursing records inconsistent with the Omaha intervention subsystem (*n* = 7).

Omaha intervention	Concept description of inconsistency	*n*
Education, guidance, counseling	Change the sweat and wet pants in time	1
Treatment and process	Diet management, low salt diet, record access, set ECG monitoring alarm authority	4
Case management	First aid knowledge, skills guidance, referral to superior medical institutions	2

### Application of Omaha problem classification scheme

The item application rate is the number of care-related problems description items in the extracted medical records/the total number of entries in the Omaha system * 100%. The Omaha problem classification scheme includes 42 health problems, 351 items describing potential/existing symptoms/signs. In this study, 37 health problems and 172 potential/existing symptoms/signs were applied in the nursing records of children with DCM, and the application rate of health problems was 88.10%. The overall application rate of potential/existing symptoms/signs was 49.00%. The specific application for each health problem is detailed in Table 4.

**Table 4:** Application of various problems and entries in the Omaha problem classification subsystem.

Field	Involved heath problem	Total items (*n*)	Entries applied (*n*)	Application rate (%)
Environment	Income	6	2	33.33
	Hygiene	12	2	16.67
	Dwelling	11	3	27.27
Social and psychology	Link to community	12	5	41.67
	Social activity	4	3	75.00
	Role shift	4	3	75.00
	Human relation	9	6	66.67
	Grief	5	1	20.00
	Mental health	18	9	50.00
	Caring/mothering	10	8	80.00
	Negligence	7	5	71.43
	Abuse	9	1	11.11
	Growth and development	3	3	100.00
Biology	Hearing	6	1	16.67
	Vision	9	4	44.44
	Language	7	2	28.57
	Oral hygiene	8	4	50.00
	Cognition	10	3	30.00
	Ache	7	7	100.00
	Consciousness	5	4	80.00
	Skin	11	7	63.64
	Neuro-musculo-skeletal function	14	7	50.00
	Respiration	10	10	100.00
	Circulation	12	12	100.00
	Digestion-hydration	12	12	100.00
	Defecation	8	6	75.00
	Urination	10	6	60.00
	Pregnancy	7	1	14.29
	Postpartum	8	2	25.00
	Infection	8	5	62.50
Health-related behaviors	Nutrition	12	7	58.33
	Sleep and rest morphology	8	2	25.00
	Physical activity	4	3	75.00
	Self-caring	10	2	20.00
	Family planning	1	1	100.00
	Caring direction	7	6	85.71
	Drug treatment program	8	7	87.50

### Application of the Omaha intervention scheme

The Omaha intervention scheme contained 4 intervention categories and 75 intervention directions. A total of 592 interventions were extracted from 76 medical records of children with DCM, including all four intervention categories. The application rate of intervention categories reached 100%, including 255 (43.07%) in education, guidance, and counseling, 215 (36.32%) in treatment and procedure, 8 (1.35%) in case management, and 114 (19.26%) in monitoring. Details were listed in Table 5.

**Table 5:** Application of the Omaha intervention subsystem intervention categories (*n* = 592).

Intervention category	Measurements (*n*)	Application rate (%)
Education, guidance, counseling	255	59.21
Treatment and process	215	27.63
Case management	8	9.21
Surveillance	114	15.79

## Discussion

Standardized nursing languages documented during care that accurately describes nursing diagnosis, interventions, and outcomes of care (18). Omaha system, one of the standardized languages, originated from community care practice in the United States. The premise of the application of this system is to match the content of clinical nursing work. Mapping is the program that explains other transactions with the same vocabulary or similar meaning (19). In the present study, we assessed the degree of conceptual consistency between the medical records and Omaha system in DCM children hospitalized in our hospital. The conceptual consistency was up to 96.19%, higher than the standard value (80%) (20). Similar findings were discovered in the published studies (21–23). It was indicated that Omaha system may be an effective tool for continuous nursing care of DCM children.

Omaha problem classification scheme focuses on four areas of nursing problems according to symptoms and signs: environment, physiology, social psychology, and health-related behaviors. It can comprehensively and intuitively reflect the health problems of patients, which enables nurses to master the rules of patients' health problems, playing an important role in practice guidance (20). There were 37 health problems involved, and their application rate is 88.1%, while the application rate of 172 related symptoms and signs is 49%, indicating that DCM children were involved in many health problems, but the related symptoms and signs were relatively concentrated, mainly in physiology, health behavior and social psychology. The specific manifestations included high care stress, anxiety, decreased cardiac output, edema, high/low blood pressure, fast/slow heart rate, arrhythmia, precordium discomfort, breathing abnormalities, cyanotic lips, water/electrolyte imbalance, poor appetite, oliguria/anuria, infection, and drug side effects. DCM has the characteristics of enormous complications, clinical diversity, and concealability, which increases the difficulty of early clinical identification, diagnosis, and treatment (24). Omaha system summarized the common nursing problems of children patients, making nurses give timely assessment and intervention in children with these potential/existing health problems, and improving the health outcome of children with heart disease (25). At the same time, it also suggests that nursing management personnel should strengthen the assessment of common problems, intervention training, and quality control of DCM children, so as to further ensure the nursing quality of the children. In this study, environmental-related nursing problems were rarely involved. The main manifestations were whether there was medical insurance and allergens in the living environment, which may be related to the current vigorous implementation of domestic medical insurance policies and routine allergy assessment. At the same time, if the hospital was set up with a unified planning and layout, the environmental factors would have little impact on hospitalized children. However, DCM children need long-term drug treatment and continuous continuation care. In community or home care, the influencing factors of environmental residence, and neighborhood/workplace safety cannot be ignored.

Omaha system can summarize the intervention mode, intervention direction, and application frequency of nursing measures for patients with the same disease, and provide the basis for the development of nursing plan, resource allocation, and continuous care (22, 23, 26, 27). This study showed that the nursing of DCM children was mainly guided by education, guidance, counseling, treatment, and procedural intervention, with an application rate of 59.21%, 27.63%, respectively. The main intervention direction was medication management, nursing care, laboratory results, care for cardiac function, safety, disease and trauma care, drug effects and side effects, diet management, care, and parenting skills, skin care, and respiratory therapy care. The results suggested that the care of DCM children featured large knowledge demand, heavy task, complex disease treatment, as well as nursing staff education, and consultation in the diagnosis and treatment process. Caregivers should be guided to provide diversified health education to meet and expand the health needs of children caregivers’ health care, improve the common treatment intervention ability, and improve the level of nursing. The application rate of case management intervention category in the care of children with DCM was the lowest, which was 1.39%. Case management aims to coordinate, encourage and refer to improve medical services (21). Due to the difficulty of diagnosis and treatment of DCM, the imbalance of existing domestic medical resources, and the imperfect referral mechanism, the application rate of the case management intervention category is low. DCM children often travel between hospitals, communities, and families. Due to the deepening of hierarchical diagnosis and treatment work, it is necessary to establish effective links and sharing among institutions. Case management, therefore, will become the trend of intervention for children with DCM. DCM Children and their families are always under heavy social pressure and mental pressure (26, 28). In this study, the application rate of nursing problems in the psychosocial field was 46.32%. However, the application of social work, counseling services, mental and emotional symptoms, signs intervention, occupational therapists, recreational treatment care, and nursing care of auxiliary professionals were lacking in the main intervention directions. The main reason is that with the deepening of the overall nursing mode, nursing staff may pay more attention to the nursing problems in the social psychological field of patients, but lack of relevant professional knowledge training. The multidisciplinary care mode for children with heart disease has not been formed, so there were few relevant nursing measures. According to the domestic and foreign guidelines, multidisciplinary team care model is recommended for DCM children in order to improve their quality of life and long-term prognosis (25, 28, 29). It was expected to strengthen the professional course training of nursing staff on social psychological nursing problems in school and in hospitals, and actively explore the application research of multidisciplinary care model in DCM children.

Although Omaha system has been widely used in disease management, nursing education, information construction, quality improvement and other nursing fields, some nursing problems and intervention direction do not need to be involved due to the popularity and universality of Omaha system concepts, such as reproductive function, sex, substance abuse and so on. On the other hand, there also were certain limitations to the Omaha system concept. From the entries of inconsistency, the system lacked some important symptoms, signs, and intervention direction, such as water and sodium retention, heart insufficiency, cardiac enlargement, heart area uplift, excessive sweating, disruption of milk intake, moan, suffocation, capacity management, low salt diet, input-output number record set electrocardiogram monitoring alarm authority, etc. The Omaha intervention system only provided intervention directions without specific intervention measures. Therefore, the application of Omaha system in the care of children's DCM needed to be modified and improved according to Chinese culture, the characteristics, and trends of children's DCM care. Under real circumstances, researchers need to use specific nursing methods by choosing common nursing problems, taking intervention measures, and combining them with the nursing information system, which can not only improve the efficiency of clinical nursing implementation, strengthen the evaluation of implementation effect and quality improvement, but also optimize the DCM child care mode, further promote the application of Omaha system in children with chronic diseases.

Herein, we first explored the feasibility of Omaha system for continuous nursing care of DCM children. Omaha system may be an effective care tool for Chinese DCM children, which may be useful to guide nurses in the continuous nursing care of DCM. There were some limitations that should be noted in interpreting our findings. First, this was a retrospective study, which shows the inherent limitations in the quality and completeness of the data available. We could not evaluate the nursing effect of the Omaha system in DCM children. Second, the limited sample size of 76 eligible DCM children with 1,392 nursing records were included. Third, the convenience sampling was carried out in this study, which may raise questions about sample representativeness and potential selection bias. Further studies utilizing more rigorous methods, such as prospective randomized controlled trials, may be necessary to fully evaluate the feasibility and effectiveness of Omaha system in nursing children with DCM.

## Conclusion

In the current study, we explored the conceptual consistency of the medical records and Omaha system. Our findings showed high conceptual consistency between the medical records and Omaha system in DCM children. Omaha system through appropriate debugging may be used as a standardized language for standardized nursing of DCM children in China, so as to promote hospital-community-family care, cross-professional, cross-regional homogeneity communication, improve the care quality of DCM children and multidisciplinary care Wmode, and promote the application of the standardized nursing language in the field of pediatrics.

## Data Availability

The raw data supporting the conclusions of this article will be made available by the authors, without undue reservation.
