# Supplementary material for: Feasibility of the Omaha system for the care of children with dilated cardiomyopathy
Source: Front Pediatr. 2023 May 30;11:1136663. doi: 10.3389/fped.2023.1136663 (PMC10267824; doi:10.3389/fped.2023.1136663)
Supplement: Supplementary file 1 [file Table1.docx]

**Table S1** Common care problems in children with DCM

| Field | Items | Specific description of the nursing problems |
| --- | --- | --- |
| Social activity | Mental health | Sadness, despair, anxiety, fear, worry about disease prognosis/drug side-effects, apathy, irritability, unpleasant activity, susceptibility to fatigue |
|  | Caring and mothering | Lower activity endurance, decreased self-care ability, low education level/advanced age of caregivers, lack of communication with parents, forced school suspension, growth retardation, nutritional disorders, high financial stress, nursing stress, and lack of nursing knowledge |
| Biological activity | Ache | Front heart area pain, front heart discomfort, front heart discomfort, palpitations, heart tiredness, chest tightness, liver area tenderness, sitting and lying position, body flexion, shortness of breath, pulse/heart rate increase, increased blood pressure, irritability, irritability, painful face, pale, sweating |
|  | Skin | Impaired skin integrity, stress damage, ecchymosis, clubbing finger |
|  | Respiration | Abnormal respiratory type, assisted breathing, mouth cyanosis/violet, ineffective respiratory clearance/inefficient, cough, abnormal sputum traits, abnormal respiratory sounds, and abnormal respiratory-assisted examination results  Reduced cardiac output, edema/edema, cyanosis, pale, jugular vein/scalp venous irritation, blood pressure/too low, arrhythmia |
|  | Circulation | Fast/too slow, arrhythmia, anterior heart pain, heart tone, heart murmur, coagulation, myocardial markers/B brain sodium peptide/serum digoxin blood concentration, electrocardiogram, heart, chest X-ray/CT findings  Nausea/vomiting, indigestion, bloating, food refusal/poor appetite, strenuous |
|  | Digestion and hydration | Nausea/vomiting, dyspepsia, abdominal distension, food refusal/ poor appetite, feeding difficulty, anemia, liver enlargement, jaundice, water/electrolyte disturbance |
| Health-related behavior | Urination | Oliguria/anuria, urine laboratory findings were abnormal |
|  | Infection | Fever, pulmonary infection, low immune function |
|  | Nutrition | Low body weight, feeding difficulties, unreasonable choice of feeding tools, lack of feeding knowledge, failure to add complementary food on time, lower than the body needs, excessive salt restriction, poor appetite /food refusal, partial food |
|  | Health direction | Failure to immunize according to the card schedule, irregular follow-up, lack of health education resources, self-medication, nonstandard drug use, failure to pay attention to the beginning, failure to seek medical treatment in time, local medical institutions lack the ability of children's specialized medical assistance, and caregivers refused relevant examinations |
|  | Medication | Failure to take medication, drug side effects (electrolyte imbalance, liver / renal impairment, digoxin poisoning performance), failure to immunize according to the card schedule, improper drug storage/dispensing |

DCM: dilated cardiomyopathy.

**Table S2** Common care measures for children with DCM

| Items | Specific description of the care problems |
| --- | --- |
| Medicine management | Rational use of drug according to doctor's advice, guidance of drug use / storage methods, high-risk drug management, centralized configuration of intravenous drug configuration center, informed consent of drug use, signing of self-provided drug responsibility letter, guidance and observation of drug efficacy and adverse reactions |
| Caring | Grading nursing, nursing risk assessment and intervention, pain assessment and intervention, heart function grading assessment, avoidance of heart failure triggers, rest and activity guidance, fluid management, condition observation and disposal, diet guidance, medication guidance, skin care, psychological care, health education |
| Laboratory result | Following the doctor's advice to assist the auxiliary inspection, auxiliary inspection of matters needing attention, health education, critical value disposal |
| Cardiac care | Assessment of cardiac function, control infusion volume/speed, monitor weight/edema changes, reduce stimulation, rest and activity guidance, administration of enhanced myocardial contractility/nutritional myocardial/vasoactive drugs/diuretics, volume management, keeping stool unobstructed, measure vital signs, and closely observe observation/laboratory results |
| Safety | Risk assessment for caring, falls and falling from bed, common risk management, high-risk management of asphyxia, nutrition, and stress injury |
| Disease/trauma care | Electrocardiogram monitoring, monitoring of vital signs, monitoring of heart rate/rhythm, monitoring of blood oxygen content, oxygen inhalation, rapid establishment of venous channels, electrical cardioversion, cardiopulmonary resuscitation |
| Effect/side effects of drugs | Review of the medication history, measurement of the pulse / heart rate before the use of the digitalis preparations, monitor of the blood drug concentration, observation of the drug efficacy and side effects, and guidance of the drug dismounting |
| Diet | Diet category/structure guidance, water and sodium control, a small number of multiple meals, and avoid overeating |
| Caring method | Feeding tools/position/respiratory regulation guidance, guidance of the prevention and emergency treatment of asphyxia, exercise rehabilitation guidance, indwelling gastric tube and care, oral care, bed defecation guidance, special disease state vaccination education, support/encourage children to participate in activities within their capacity |
| Skin | Reasonable turn over according to the severity of cardiac function, avoid long-term compression of local tissue, strengthen nutritional support, keep the skin clean and dry, monitor edema changes, avoid iatrogenic skin injury, local use of decompression patch |
| Respiration | Observation of the frequency/morphology of breathing, monitoring of the blood oxygen content, oxygen inhalation, keep the airway patency, observation of the frequency/nature of cough, guide the atomization inhalation, guidance of the effective cough/sputum discharge method, observation of the amount/color/nature of sputum, guidance of the measures to prevent respiratory tract infection, and compliance with doctor's advice and rational drug use |

DCM: dilated cardiomyopathy.
